# Effect and Concern of Breastfeeding in Infants

**DOI:** 10.14789/jmj.JMJ24-0003-R

**Published:** 2024-07-26

**Authors:** HIROMICHI SHOJI

**Affiliations:** 1 Department of Pediatrics Medicine, Juntendo University Faculty of Medicine, Tokyo, Japan; 1 Department of Pediatrics Medicine, Juntendo University Faculty of Medicine, Tokyo, Japan

**Keywords:** human milk, oxidative stress, infants

## Abstract

Human breast milk is considered the optimal source of nutrition for infants and is recommended as the exclusive nutrient source for term infants during the first six months of life. Existing evidence strongly supports the direct benefits of breastfeeding, encompassing benefits for nutrition, gastrointestinal function, and protection against acute illness in both term and preterm infants. Previously, we demonstrated a notable reduction in a urinary marker of oxidative DNA damage in breastfed term and preterm infants compared to formula-fed infants. While long-term benefits of breastfeeding on neurodevelopmental outcomes and adult health have been reported, the effects may be relatively modest and limited.

## Introduction

Breastfeeding provides optimum support for infant and maternal health, and is recommended in both industrialized and developing countries as the exclusive nutrient source for term infants during the first six months of life^1)^. In the Global Nutrition Targets for 2025, the World Health Organization recommends increasing the rate of exclusive breastfeeding up to at least 50% among infants less than six months^2)^. Human breast milk offers nutrients with high bioavailability and in sufficient qualities to support infant growth, and this practice is recommended to be continued alongside the introduction of complementary foods after the initial six months^1)^. Various factors, such as the duration of lactation, gestational length, maternal health, genotype, and diet, influence the composition of milk and the production of colostrum, transitional, and mature milk^3)^. Colostrum contains a high concentration of whey proteins and exhibits lower levels of lactose and fat compared to mature milk^3, 4)^. Colostrum includes high concentrations of bioactive compounds, including secretory immunoglobulin A (sIgA) and lactoferrin^5)^. sIgA plays a crucial role in protecting the intestinal epithelium from enteric toxins and pathogenic microorganisms. Through a process known as immune exclusion, sIgA facilitates the clearance of antigens and pathogenic microorganisms from the intestinal lumen by blocking their access to epithelial receptors and entrapping them in mucus^6)^. This review aimed to summarize the existing literature on the health benefits of breastfeeding in term and preterm infants.

## Macronutrients in human milk

The macronutrients of human milk and their contributions to the total energy intake of breastfed infants are derived from carbohydrates (45%), fats (44%) and proteins (8%) at one month of age^7)^.

### Proteins and nonprotein nitrogen

The protein content of human milk is at its peak during the production of colostrum (approximately 15 to 20 g/L) and gradually decreases in mature milk from the second to the sixth or seventh month of lactation, reaching approximately 10 g/dL^3, 4)^. Human milk contains a heterogeneous mixture of over 400 casein, whey, and mucin proteins and peptides that provide nutrition, antimicrobial and immunomodulatory activities, and stimulate nutrient absorption^8)^. Proteins are essential for infant growth, with specific proteins (e.g., lactoferrin and α-lactalbumin) acting as carriers of nutrients, promoting gut development (e.g., growth factors and insulin), aiding nutrient absorption (e.g., bile salt-stimulated lipase, amylase, and α-antitrypsin), or exhibiting immune and antimicrobial activity (e.g., lactoferrin and sIgA)^9)^. Colostrum has a whey-predominant composition (90:10 whey/casein ratio) and elevated concentrations of growth factors, sIgA, lactoferrin, and lysozyme compared to mature milk. The quantity and quality of milk proteins exert a significant influence on infant growth and body composition. High protein intake during infancy activates the insulin-like growth factor-I axis and has been associated with increased weight gain and a higher risk of obesity later in life^10)^. The total nitrogen content in human milk follows a similar pattern, being highest in colostrum (3.0 g/L) and decreasing in mature milk (1.9 g/dL)^3, 4)^. Nonprotein nitrogen, encompassing urea, creatinine, nucleotides, free amino acids, and peptides comprise 25% of the total nitrogen in human milk. Nucleotides, considered conditionally essential nutrients, modulate enzyme activities and promote the development and maturation of the gastrointestinal and immunological systems^3)^.

### Carbohydrates

The principal sugar in human milk is lactose, a disaccharide with a concentration of approximately 6.7 g/dL. This concentration surpasses that found in the milk of other species and underscores its significance in meeting the nutritional requirements of the brain^3)^. Lactose serves as an important source of galactose, essential for the development of the central nervous system. Other major carbohydrates found in human milk are oligosaccharides, ranging from 2.1 g/dL in colostrum to 1.3 g/dL in mature milk^11)^. Over 200 acidic or natural oligosaccharides have been identified in human milk, with their composition influenced by maternal genetics^12, 13)^. While oligosaccharides are not digestible, they function as prebiotics that serve as metabolic substrates for beneficial bacteria, including *Bifidobacteria* and *Bacteroides* species. Additionally, oligosaccharides modulate infant mucosal and systemic immune functions^14)^.

### Lipids

Lipids, present as an emulsion, contribute 40-50% of the total energy of human milk^7)^. Colostrum contains lipid concentrations of 1.5-2.0 g/dL, while mature milk exhibits 3.5-4.8 g/dL. Approximately 98% of milk lipids are secreted as triglycerides, serving as a crucial source of essential nutrients such as polyunsaturated fatty acids (PUFAs), lipid-soluble vitamins, complex lipids, and bioactive compounds. The remaining lipid content consists of diglycerides, monoglycerides, free fatty acids, phospholipids, and cholesterol^7)^. The properties of triglycerides are determined by their fatty acid composition, with minimal non-estrified fatty acids present in human milk. Triglycerides account for 88% of the fat, with approximately 43% saturated fatty acids, 35% cis-monosaturated, 1% to 7% trans- monounsaturated, and 20% PUFAs (19% n-6 and 1% n-3)^15)^. Although the lipid content is influenced by the mother’s diet, palmitic acid is the most predominant saturated fatty acid, commonly found in the sn-2 position. This positioning enhances absorption and improves calcium absorption. Most infant formulas contain triglycerides with palmitic acid in the sn-1 and sn-3 positions. Pancreatic lipase selectively hydrolyzes triglycerides in these positions, producing two free fatty acids and a monoglyceride. The 2-monopalmitin produced during the digestion of human milk fat is readily absorbed, while the free palmitate formed during the digestion of formula milk may combine with calcium to form soaps and be lost in the feces^16)^.

The long-chain polyunsaturated fatty acids (LCPUFAs) in human milk are influenced by the mother’s diet, with those possessing more than 20 carbons and 2 or more double bounds comprise only about 2% of the total fatty acids in breast milk^17)^. Human milk contains n-3 and n-6 LCPUFAs, such as arachidonic acid (AA, 20:4, n-6) and docosahexaenoic acid (DHA, 20:4 n-3). A systematic review reported worldwide concentrations (by weight) of 0.47 ± 0.13% for AA and 0.32 ± 0.22% for DHA. The DHA content tends to be lower and more variable based on the mother’s diet^18, 19)^. The importance of dietary intake of AA and DHA for visual and cognitive development, particularly in preterm infants^20)^. Consequently, LCPUFAs are now routinely supplemented in preterm and term infant formulas.

## Benefits of breastfeeding

### Direct effects

The antimicrobial and immunomodulatory properties of human milk support the immature neonatal immune system and inhibit the movement of pathogens across the gastrointestinal barrier^21)^. Notably, breastfed infants exhibit a more stable and less variable intestinal microbiota compared to formula- fed infants, with over twice the number of bacterial cells^22)^. Human milk contains various factors that inhibit inflammation or stimulate antibody production, including platelet-activating factor acetylhydrolase (PAF-AH), interleukins, and transforming growth factor^21)^. Colostrum contains high concentrations of bioactive compounds such as sIgA, lactoferrin, and leukocytes^5)^. Moreover, specific components of human milk, such as insulin-like growth factor, insulin, and epidermal growth factor, actively promote gastrointestinal growth, motility, and maturation, and may be protective against disease^23)^.

### Antioxidant properties

**Figure 1:**
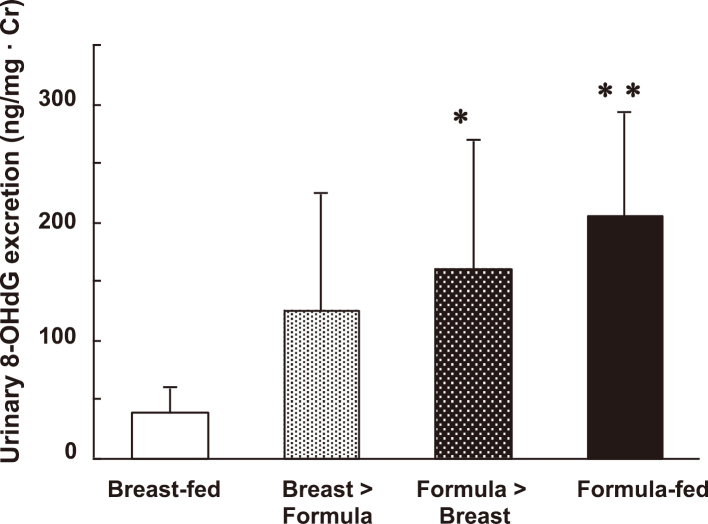
Urinary 8-hydroxydeoxyguanosine (8-OHdG) in one-month-old term infants Values are expressed as mean ± SD *p < 0.05, **p < 0.01 compared with values of the breastfed group

Human milk is rich in various antioxidant enzymes, including catalase, glutathione peroxidase, and superoxide dismutase. Additionally, it contains essential cofactors such as copper (Cu) and zinc (Zn); vitamins A, C, and E; and binding proteins such as lactoferrin^24)^. In contrast, infant formulas lack antioxidative enzymes^25)^, but compensate with the inclusion of higher amounts of vitamin supplements compared to human milk to compensate for the reduced bioavailability. Therefore, evaluating the overall antioxidant capacities of human milk versus infant formulas is challenging; however, it is likely that human milk is favorable in this regard^26)^. A previous study reported significantly lower urinary excretion of 8-hydroxy-2′-deoxyguanosine, a marker of oxidative DNA damage, in breastfed term infants compared to formula-fed term infants (Figure 1)^27)^. This difference may be attributed to the presence of antioxidants in human milk, which are effectively transferred through the relatively porous neonatal intestine.

## Breastfeeding and associated outcomes

### Short-term health outcomes

Human milk provides direct benefits that include supporting gastrointestinal function, enhancing host defense, and preventing acute illnesses such as acute otitis media during breastfeeding. The protective role of breastfeeding against infections is considered one of its most important health benefits^1, 28)^. A review by Kramer and Kakuma supported exclusive breastfeeding for six months to prevent infection^29)^. The Promotion of Breastfeeding Intervention Trial (PROBIT) reported relative risks of 0.67 (95% confidence interval [CI]: 0.46-0.97) for acute gastroenteritis episodes and 0.75 (95% CI: 0.60-0.94) at 12 months of age, comparing 6-7 months to 3-4 months of exclusive breastfeeding. Systematic reviews from 2016 emphasized a strong protective effect of exclusive breastfeeding for the first six months of life, resulting in an 88% reduction in infectious disease mortality compared to non-breastfed infants^30, 31)^. Three studies conducted in low- and medium-income countries found that the absence of breastfeeding was associated with an increased risk of mortality. The protective effect of breastfeeding demonstrated a dose-dependent relationship, with a 78% reduction in the risk of death associated to predominant breastfeeding and a 48% reduction associated with partial breastfeeding^31)^. In resource-rich countries, breastfed infants exhibit a lower attack rate of acute illnesses compared to formula-fed infants^32)^. In addition, a meta-analysis found that exclusive breastfeeding has been associated with a 36% (95% CI: 19-49) reduction in incidence of sudden infant death syndrome^33)^. However, the evidence of a protective effect of breastfeeding against eczema or food allergy was found to be less strong^30)^. In 2020, Sakihara, et al. reported on the results of the Strategy for Prevention of Milk Allergy by Daily Ingestion of Infants Formula in Early Infancy (SPADE) study, which demonstrated that the continuous daily ingestion of about 20 mL of formula milk between 1 and 2 months of age prevented the development of cow’s milk allergy^34)^. Meanwhile, another study found a modest inverse association between breastfeeding and circulating insulin levels in infancy^35)^. Moreover, human milk contains hormones such as leptin, adiponectin, resistin, and ghrelin, actively contributing to the regulation of energy balance and glucose homeostasis^36)^.

### Long-term health outcomes

Breastfeeding has been associated with a reduction in the risk of hypertension^37, 38)^, obesity^39, 40)^, and insulin resistance^41, 42)^ during adulthood, all of which contribute to metabolic syndrome (MS). While a definitive long-term benefit of human milk versus formula milk in reducing the incidence of MS remains unclear, recent findings from a meta- analysis found that longer exposure to breastfeeding was associated to a 35% (95% CI: 14-51) reduction in the risk of developing type II diabetes^43)^. However, a protective effect of breastfeeding against hypertension and/or hypercholesterolemia was not observed^43)^. A nationwide longitudinal survey conducted in Japan between 2001 and 2009 found that exclusive breastfeeding decreased the risk of overweight (adjusted odds ratio [OR]: 0.85, 95% CI: 0.69-1.05) and obesity (adjusted OR: 0.55, 95% CI: 0.39-0.78)^44)^. Moreover, a previous meta-analysis indicated that increased breastfeeding duration was associated with a 26% reduction in the odds of overweight or obesity, and this effect was consistent across income levels^30)^. However, not all studies have consistently found an association between breastfeeding and a lower risk of overweight or obesity^45, 46)^. In terms of childhood leukemia, a meta-analysis of 18 case-control studies revealed that breastfeeding reduced the overall risk by 20% (OR: 0.80, 95% CI: 0.82-0.90)^47)^. Breastfeeding has consistently demonstrated positive effects on cognitive outcomes. A previous study indicated that exclusively and more breastfed infants had a mean intelligence quotient 3.4 points higher (95% CI 2.3-4.6) than that of never or less breastfed infants^48)^. Another meta-analysis confirmed higher cognitive function (3.2 points) in exclusively and partially breastfed infants compared to formula-fed infants, with improved scores persisting throughout childhood and adolescence. The benefit was particularly pronounced in low birth weight infants compared to those with normal birth weight^49)^. However, when controlling for maternal IQ and other confounders, the effect was small (0.52 points) and not significant^50)^. In the PROBIT, the only prospective randomized study, the intervention group exhibited significantly higher intelligence scores and Thatcher’s rating^51)^.

## Benefits for preterm infants

**Figure 2:**
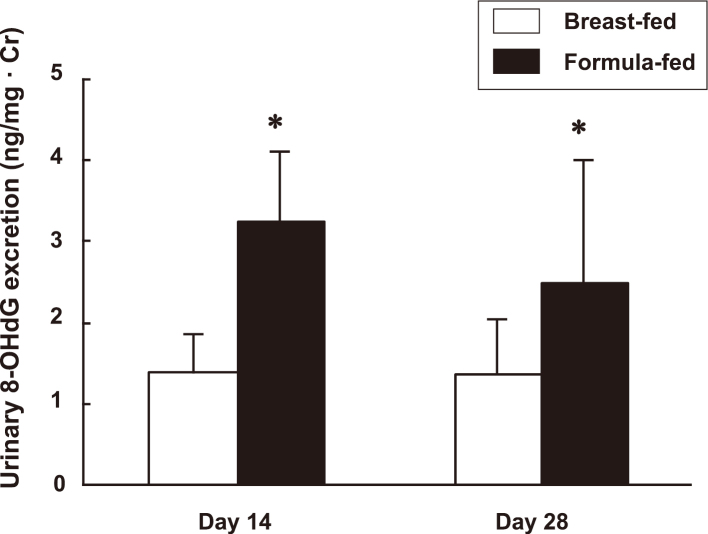
Change in urinary 8-hydroxydeoxyguanosine (8-OHdG) excretion at 14 and 28 days of age in breastfed and formula-fed very low birthweight infants Values are expressed as mean ± SD *p < 0.01 compared with breastfed infants

Human milk is recommended for preterm infants because of its association with a reduced incidence of necrotizing enterocolitis (NEC)^52)^ and improved IQ scores^53)^. Breastfed preterm infants exhibit a 6- to 10-fold lower risk of NEC compared to formula-fed infants^54)^. While the exact cause is yet to be determined, factors such as immunoglobulins, PAF- AH, PUFAs, epidermal growth factors, or interleukin-10 (IL-10) in human milk^55)^, and the colonization of the intestine with *Bifidobacteria* and *Lactobacilli* species may have been involved. Breastfeeding is also associated with a decreased incidence of oxidative stress-related illnesses in preterm infants, including respiratory disease^56)^ and retinopathy of prematurity^57)^. A previous study demonstrated that urinary 8-OHdG excretion at 14 and 28 days of age was significantly lower in breastfed preterm infants compared to formula-fed preterm infants (Figure 2)^58)^. However, the nutrient content of both term and preterm breast milk is insufficient to meet the needs of infants weighing less than 1,500 g, necessitating supplementation with a human milk fortifier^52)^. The benefits of breastfeeding for preterm infants extend beyond the neonatal intensive care unit (NICU), with fewer hospital readmissions for illnesses in the year following NICU discharge^59, 60)^. Extremely preterm infants receiving a higher proportion of human milk in the NICU demonstrated significantly increased mental, motor, and behavior rating scores at 18 and 30 months of age^59, 60)^.

## Conclusions

Breastfeeding is unequivocally endorsed by all medical professional organizations and public health authorities. Current evidence strongly supports the direct benefits of breastfeeding across various dimensions, encompassing nutrition, gastrointestinal function, and protection against acute illness, both in term and preterm infants. Long-term benefits of breastfeeding on neurodevelopmental outcomes and adult health have been reported; however, the magnitude of these effects may be modest and limited.

## Funding

No funding was received.

## Author contributions

The author read and approved the final manuscript.

## Conflicts of interest statement

The author declares that there are no conflicts of interest.
